# Simulation-Based Training – Evaluation of the Course Concept “Laparoscopic Surgery Curriculum” by the Participants

**DOI:** 10.3389/fsurg.2016.00047

**Published:** 2016-08-09

**Authors:** Ferdinand Köckerling, Michael Pass, Petra Brunner, Matthias Hafermalz, Stefan Grund, Joerg Sauer, Volker Lange, Wolfgang Schröder

**Affiliations:** ^1^Department of Surgery, Center for Minimally Invasive Surgery, Vivantes Endoscopic Training Center, Academic Teaching Hospital of Charité Medical School, Vivantes Hospital, Berlin, Germany; ^2^Department of General, Visceral- and Minimally Invasive Surgery, Arnsberg Hospital, Arnsberg, Germany; ^3^Professional Association of German Surgeons (BDC), Berlin, Germany

**Keywords:** simulation-based training, laparoscopic surgery curriculum, skills in laparoscopic surgery, young surgeons, simulation-based courses

## Abstract

**Introduction:**

The learning curve in minimally invasive surgery is much longer than in open surgery. This is thought to be due to the higher demands made on the surgeon’s skills. Therefore, the question raised at the outset of training in laparoscopic surgery is how such skills can be acquired by undergoing training outside the bounds of clinical activities to try to shorten the learning curve. Simulation-based training courses are one such model.

**Methods:**

In 2011, the surgery societies of Germany adopted the “laparoscopic surgery curriculum” as a recommendation for the learning content of systematic training courses for laparoscopic surgery. The curricular structure provides for four 2-day training courses. These courses offer an interrelated content, with each course focusing additionally on specific topics of laparoscopic surgery based on live operations, lectures, and exercises carried out on bio simulators.

**Results:**

Between 1st January, 2012 and 31st March, 2016, a total of 36 training courses were conducted at the Vivantes Endoscopic Training Center in accordance with the “laparoscopic surgery curriculum.” The training courses were attended by a total of 741 young surgeons and were evaluated as good to very good during continuous evaluation by the participants.

**Conclusion:**

Training courses based on the “laparoscopic surgery curriculum” for acquiring skills in laparoscopy are taken up and positively evaluated by young surgeons.

## Introduction

The term “learning curve” as currently employed in surgery means that inexperienced surgeons have not only a longer operating time but also a higher complication rate (1). Mastery of the learning curve in surgery can no longer be merely left to “trial and error” in routine clinical practices but, instead, calls for the development, definition, and introduction of models suitable for training surgeons without presenting any higher risk to patients (1). Simulation-based training courses are one such model (1).

The learning curve in laparoscopic surgery is much longer than in open surgery. In the literature, the learning curve for laparoscopic cholecystectomy is given as 30 procedures (2, 3), for endoscopic inguinal hernia surgery as 60 procedures (4), for laparoscopic gastric bypass as 100 procedures (5), and for laparoscopic colorectal surgery as 88–152 procedures (6). This is thought to be due to the more exacting demands made on the surgeon’s skills (7). Among the factors militating against rapid acquisition of skills in laparoscopic surgery are the low number of cases suitable for teaching operations, difficulties with the video-eye-hand coordination, altered perceptions of depth, and laparoscopic suturing (8). This means that, often, even after completion of specialist surgical training, some surgeons have shortcomings when it comes to laparoscopic suturing techniques, bimanual coordination, and mastery of challenging anatomic situations (9).

Therefore, the question raised at the outset of training in laparoscopic surgery is how such skills, i.e., the skills and competencies to perform laparoscopic surgery, can be acquired by undergoing training outside the bounds of clinical activities to try to shorten the learning curve.

In a systematic review, Zendejas et al. (7) demonstrated that laparoscopic techniques can be learned more effectively in a simulation-based training course compared with when learning such techniques only during clinical training. Training on expensive virtual reality trainers is no better than when using the more favorably priced pelvic trainers and boxes with porcine organs models from abattoirs (7, 10).

Likewise, a Cochrane review identified advantages for acquiring skills in laparoscopic surgery by first participating in simulation-based training courses on pelvic trainers (11). Simulation-based training helps to shorten the operating time and enhance the ability to implement surgical techniques. The skills learned in training courses can be immediately applied for the patient in the operating room (12–15).

In a prospective randomized trial on learning the total extraperitoneal patch plasty (TEP) technique in endoscopic inguinal hernia surgery, Zendejas et al. (16) demonstrated that surgeons who had undergone such simulation-based training had significantly shorter operating times, better performance scores, and fewer intraoperative and postoperative complications than those surgeons who had not taken part in such a training course.

Based on evidence-based data, it is urgently recommended that young surgeons in training as general and visceral surgeons take part in such training courses. Below are now described the experiences gained in Germany with the introduction of a curricular concept for simulation-based training in minimally invasive surgery, which was offered in parallel to the normal specialist surgical training program.

## Methods

Based on the evidence presented above, the board of directors (M. Strik, Berlin, K. Ludwig, Rostock, R. Bittner, Stuttgart, W. Schwenk, Hamburg, M. Walz, Essen, Ferdinand Köckerling, Berlin) of the Minimally Invasive Surgery Working Group (CAMIC) of the German Society of General and Visceral Surgery (DGAV), in 2011, adopted the “laparoscopic surgery curriculum” as a recommendation for the learning content of systematic training courses in laparoscopic surgery.

The curricular structure provides for four 2-day training courses with an interrelated content and with each course focusing additionally on specific topics of laparoscopic surgery. The following key courses are recommended:

Course I: fundamentals of minimally invasive surgery and laparoscopic cholecystectomy (Table 1)
Course II: endoscopic hernia surgery [TEP, transabdominal preperitoneal patch plasty (TAPP), laparoscopic intraperitoneal onlay mesh (lap IPOM), and laparoscopic fundoplication] (Table 2)
Course III: laparoscopic suturing, knot-tying, clipping, stapling, laparoscopic hemostasis, laparoscopic appendectomy, adhesiolysis, stomach wedge resection and gastroenterostomy, and Roux-Y anastomosis (Table 3)
Course IV:laparoscopic colorectal surgery, rectopexy, sigmoid and rectal resection, total mesorectal excision (TME), right hemicolectomy and stoma placement, and intraabdominal intestinal resection (Table 4).

**Table 1:** **Course I content**.

Fundamentals of minimally invasive surgery	
Laparoscopic Cholecystectomy	
Target group: year 1– 2 of specialist surgical training	
Instruments and OR techniques	Access routes, exploration, and dissection
Video-endoscopic equipment (camera, light source, CO_2_ insufflation, irrigation-suction system, image and video documentation, monitor, etc.) Setting up the video-endoscopic equipment in the operating room Current and ultrasound for dissection and hemostasis Trocars Instruments Standardized exercises on the pelvic trainers (e.g., Lübeck toolbox)	Safe access routes Trocar placement (method, complications, trocar selection, etc.) Generation of pneumoperitoneum Physiology of pneumoperitoneum Monoport vs. several trocars, minitrocars Control of access complications Appropriate adjustment of the video-endoscopic equipment Cleaning the optics Exploratory laparoscopy Taking biopsies Blunt and sharp dissection Hemostasis techniques
**Perioperative management**	**Laparoscopic cholecystectomy**
Preoperative patient preparation – Bladder emptying – Thrombosis prophylaxis – Antibiotic prophylaxis – Discontinuation of platelet aggregation inhibitors – Patient information, etc. Patient positioning Avoidance of damage from incorrect positioning Positioning the OR team	Anatomy of the gallbladder and bile ducts Dissection of Calot’s triangle Clipping of the cystic artery and cystic duct Withdrawal of the gallbladder from the gallbladder bed Gallbladder retrieval Hemostasis of gallbladder bed Fundus first technique Drain placement Management of laparoscopic cholecystectomy complications

**Table 2:** **Course II content**.

Endoscopic hernia surgery [transabdominal preperitoneal patch plasty (TAPP), total extraperitoneal patch plasty (TEP), laparoscopic intraperitoneal onlay mesh (Lap. IPOM)], laparoscopic fundoplication	
Target group: year 3–4 of specialist surgical training	
**Fundamentals of hernia surgery**	
Anatomy of the groin, abdominal wall, and esophageal hiatus Classification of hernias Tailored approach in hernia surgery Learning curve Mesh materials for hernia surgery Pros and cons of individual mesh materials Biocompatibility of meshes Different mesh fixation techniques (suture, tackers, glue) Perioperative preparation	
**TAPP – transabdominal preperitoneal patch plasty**	**IPOM – intraperitoneal onlay mesh**
Patient positioning and OR team positioning Trocar placement Dissection techniques Dissection extent Procedure for direct hernia Procedure for indirect hernia Procedure for bilateral hernia Procedure for recurrence Procedure for lipoma Mesh insertion Mesh placement Mesh fixation Peritoneal closure Problem management	Indications Preoperative diagnosis Patient positioning and OR team positioning Trocar placement Adhesiolysis Defect repair Mesh insertion Transfascial mesh fixation Mesh fixation with suture Mesh fixation with tackers Problem management
**TEP – total extraperitoneal patch plasty**	**Laparoscopic fundoplication**
Patient positioning and OR team positioning Trocar placement Creation of the extraperitoneal space Dissection techniques Dissection extent Procedure for direct hernia Procedure for indirect hernia Procedure for bilateral hernia Procedure for recurrence Procedure for lipoma Mesh insertion Mesh placement Mesh fixation Problem management	Indications Preoperative diagnosis Patient positioning and OR team positioning Trocar placement Transection of the short gastric vessels Hiatoplasty with and without mesh Creation of a Toupet or Nissen fundoplication Problem management

**Table 3:** **Course III content**.

Laparoscopic suturing, Knot-Tying, clipping, stapling, laparoscopic hemostasis, laparoscopic appendectomy, adhesiolysis, stomach wedge resection and gastroenterostomy, Roux-Y anastomosis	
Target group: year 4–5 of specialist surgical training	
Laparoscopic suture, knot-tying, clipping, and stapling techniques	Laparoscopic stapling techniques
Laparoscopic suture materials Laparoscopic needle holders and instruments Laparoscopic knot-tying techniques Laparoscopic single button suture and continuous suture Using clips for suturing Oversewing slip suture rows Intra- and extracorporeal knot-tying techniques Using knot pushers Using Roeder slings Problems with laparoscopic suturing Strengths and weaknesses of various clips Appropriate use of clips Metal clips vs. absorbable clips	Laparoscopic clipping and stapling techniques Organ resection with stapling techniques Control of complications after using stapling devices for organ resection (bleeding, defect, hypoperfusion, etc.) Tissue reinforce on using stapling techniques
**Laparoscopic hemostasis**	**Advanced laparoscopic surgical techniques**
Hemostasis with clips Laparoscopic use of fibrin glue for hemostasis Using liquid and collagen-bound fibrin glue Application systems for fibrin glue Using starch powder for hemostasis Suture vs. clip vs. fibrin glue vs. starch powder for hemostasis. When which technique?	Laparoscopic anastomosis techniques for the stomach and small intestine Suturing the insertion site on using stapling instruments for anastomosis Laparoscopic gastroenterostomy Laparoscopic Roux-Y anastomosis Management of complications related to stomach and small intestine anastomosis (bleeding, defect, hypoperfusion, etc.)

**Table 4:** **Course IV content**.

Laparoscopic colorectal surgery, rectopexy, sigmoid and rectal resection, total mesorectal excision (TME), hemicolectomy right, stoma placement. intraabdominal intestinal resection	
Target group: year 5–6 of specialist surgical training	
**Fundamentals of laparoscopic colorectal surgery**	
Fundamentals of anastomosis Intestinal preparation Team building Learning curve Particularities of oncologic indications	
**Laparoscopic rectopexy**	**Laparoscopic right hemicolectomy**
Indications Preoperative diagnosis Patient positioning and OR team positioning Trocar placement Ureter exposure Dissection techniques Extent of rectum mobilization Rectopexy technique Problem management	Indications Preoperative diagnosis Patient positioning and OR team positioning Trocar placement Dissection techniques Extent of lymph node dissection Intracorporeal vs. extracorporeal intestinal resection Specimen retrieval Intracorporeal vs. extracorporeal anastomosis Drainage Problem management
**Laparoscopic sigmoid and rectal resection**	**Laparoscopic stoma placement**
Indications Preoperative diagnosis Preoperative marking of potential stoma position Patient positioning and OR team positioning Trocar placement Ureter exposure Dissection techniques Resection extent Total/partial mesorectal excision Transection of the inferior mesenteric artery Mobilization of the left colon flexure Intestinal resection, intraabdominal Mini-laparotomy for specimen retrieval Preparation of anastomosis Anastomosis technique Leakage test Drainage Protective stoma Problem management	Indications for ileostomy, transversostomy, and sigmoidostomy Preoperative marking of placement site Differences in technical approaches Problem management

Based on that recommendation, since 2012, the Federal Association of German Surgeons (BDC) in collaboration with the CAMIC and DGAV have been running regular simulation-based training courses at the Vivantes Endoscopic Training Center of the Department of Surgery – Visceral and Vascular Surgery – of the Vivantes Hospital Berlin (Medical Director: Prof. Dr. med. Ferdinand Köckerling).

The recommendation is that young surgeons attend the training courses in the following order: “laparoscopic cholecystectomy” course in year 1–2; “endoscopic hernia surgery (TEP, TAPP, lap. IPOM)” course in year 3–4; course; “laparoscopic suturing, knot-tying, clipping, stapling, laparoscopic hemostasis, laparoscopic appendectomy, adhesiolysis, stomach wedge resection and gastroenterostomy, and Roux-Y anastomosis” course in year 4–5; and the “Laparoscopic colorectal surgery” course in year 5–6. Currently, there is no evaluation of the participants through implementation of a score to get permission for the next course level.

**Figure 1:**
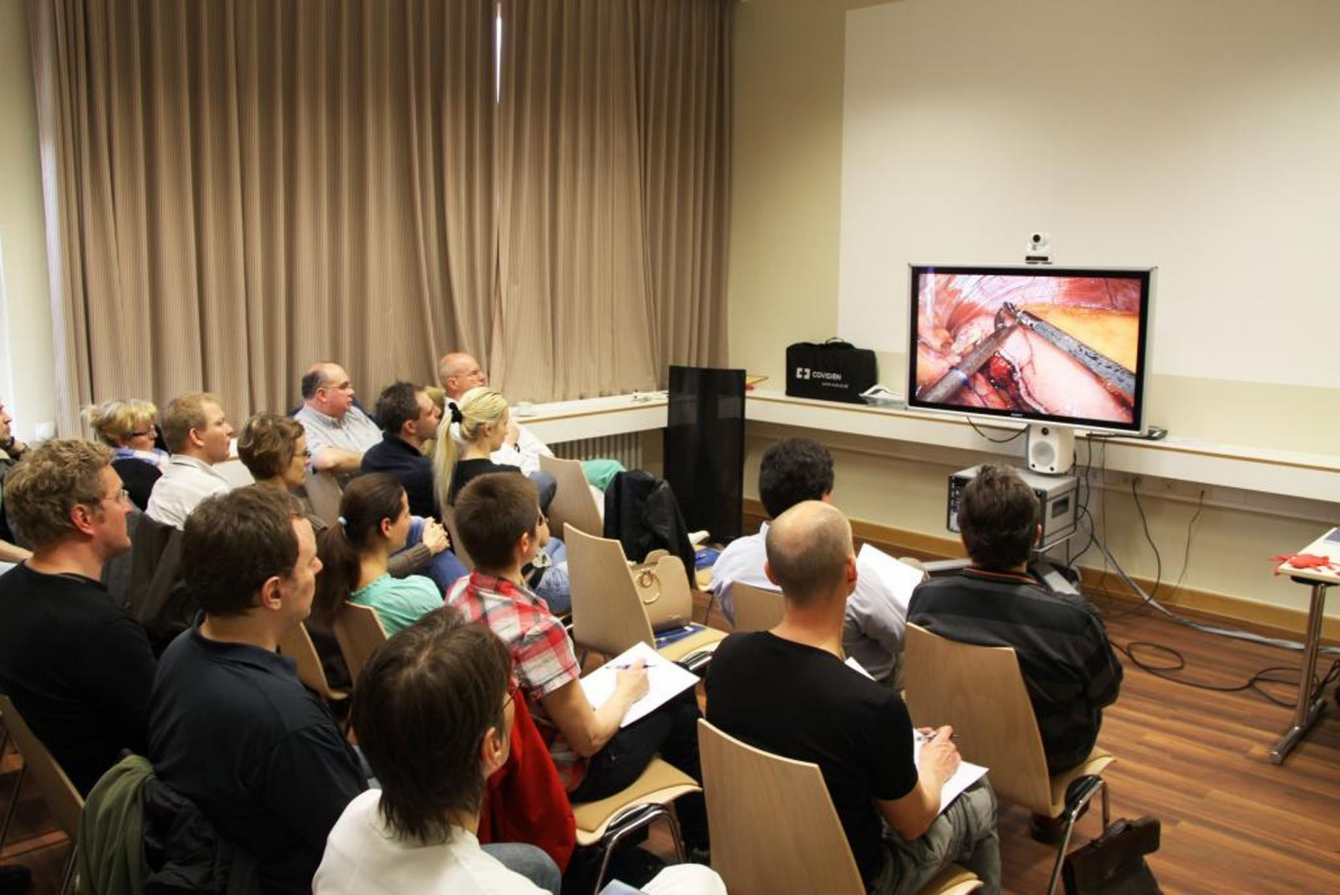
**Live transmission from two operating rooms to the lecture room**.

**Figure 2:**
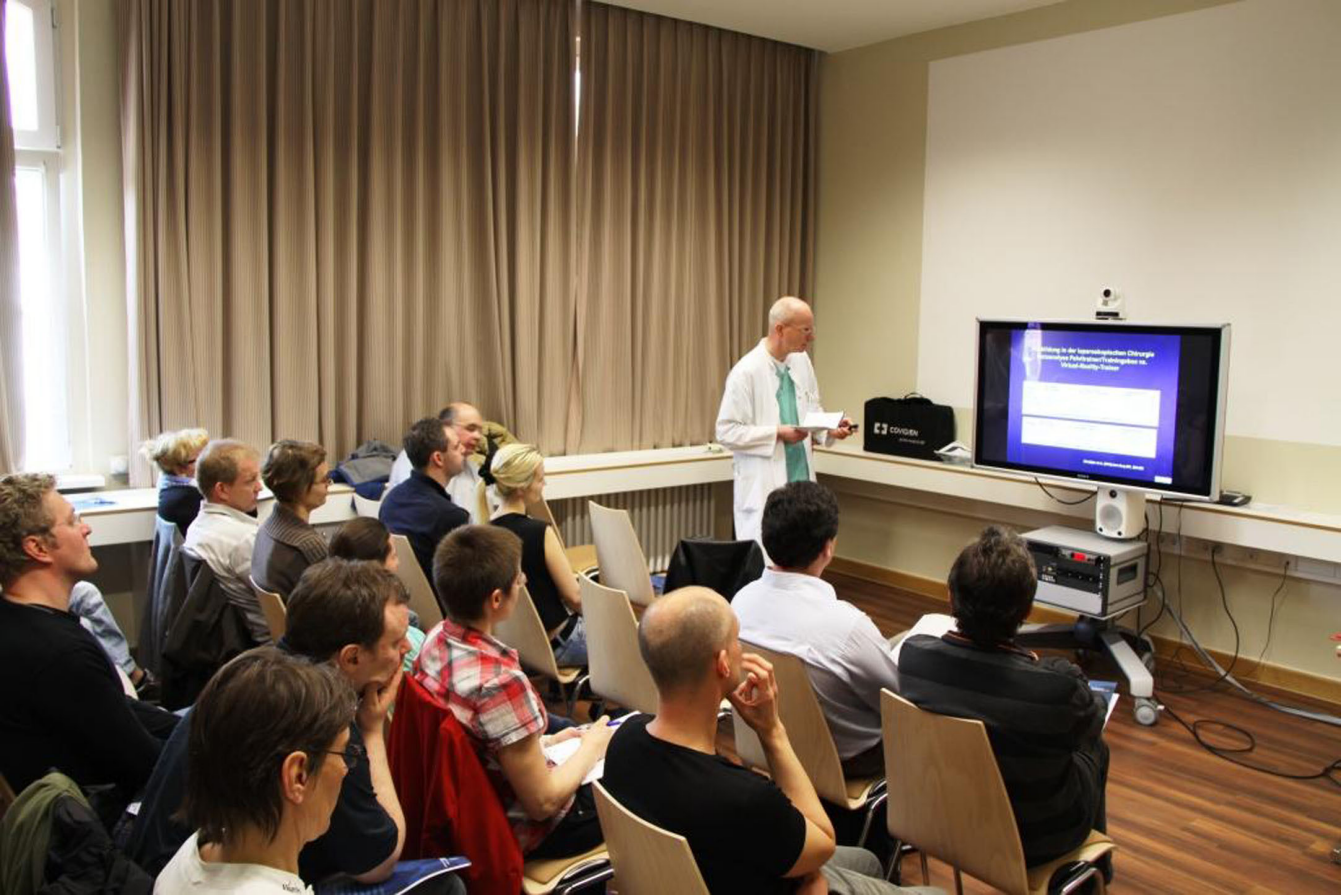
**Lectures on specific key topics**.

**Figure 3:**
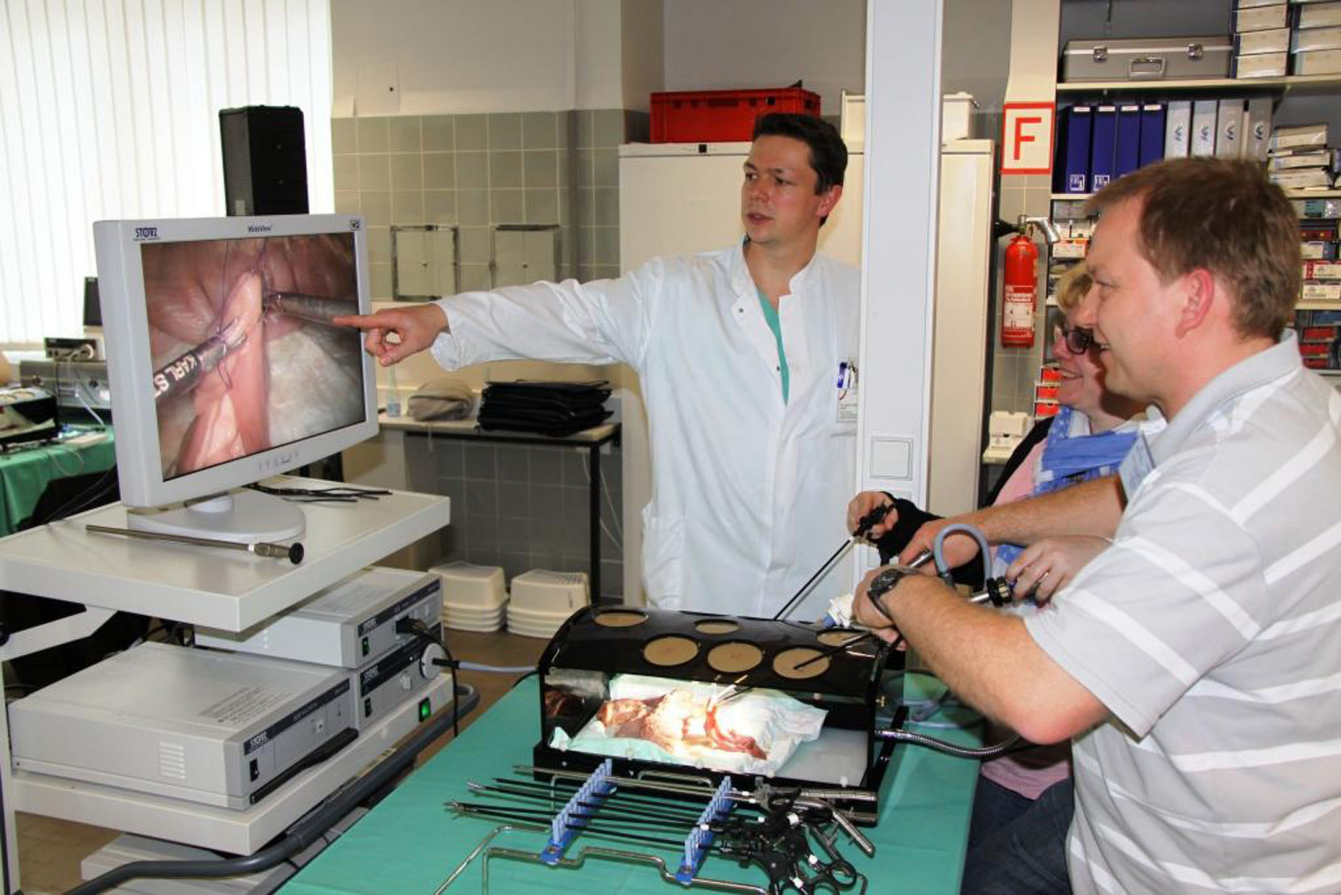
**Practical training on the bio simulator with (porcine) organs from the abattoir or chickens from the supermarket with assistance from experienced laparoscopic surgeons**.

**Figure 4:**
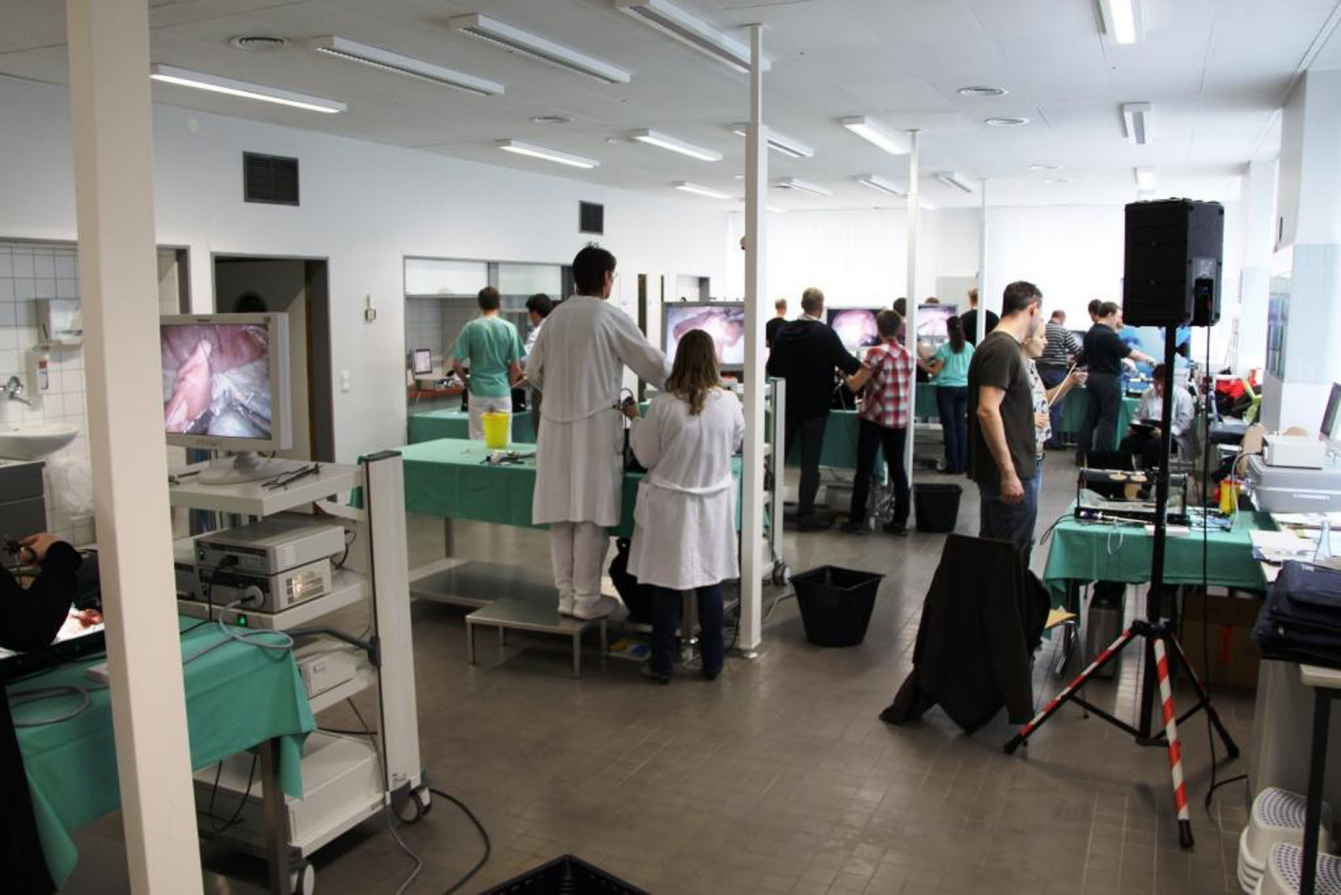
**In the Vivantes Endoscopic Training Center, up to 24 young surgeons can be trained simultaneously at 12 fully equipped working places workstations**.

The course content is imparted to participants based on live operations from two operating rooms at the Vivantes Hospital Berlin (Figure 1) and lectures (Figure 2). But, the key element is the training units carried out on bio simulators (Figure 3), which give course attendees the chance to thoroughly practice all manual skills using porcine organ models from an abattoir or chickens from the supermarket. To that effect, the same video-endoscopic equipment, as used in the operating room, is available (Figure 3). The course trainers are available to assist the attendees throughout. The bio simulators confront trainees with a situation that mimics that which they have to master in an actual surgical setting. This practical test serves to make each participant aware of his/her technical shortcomings in performing surgery, which must now be overcome. Since participation in all four training courses is mandatory for attainment of the “laparoscopic surgery curriculum” overall certificate, the progress made by individual trainees can be well monitored over the years. Since 12 fully equipped working places traineeships are available at the Vivantes Endoscopic Training Center, thanks to the support from the firms Storz and Medtronic, up to 24 colleagues can participate in each training course (Figure 4).

## Results

Between 1st January, 2012 and 31st March, 2016, a total of 36 training courses were conducted at the Vivantes Endoscopic Training Center in accordance with the “laparoscopic surgery curriculum.” The training courses were attended by a total of 741 young surgeons, and each course was evaluated on completion. Attendees were asked to evaluate the course in terms of its learning content, scope of theoretical presentations, didactic concept, duration of exercises, quality of the live operations, and overall assessment. Responses were graded as follows: 1 (very good), 2 (good), 3 (satisfactory), 4 (sufficient), 5 (deficient), and 6 (insufficient). In general, participants evaluated the courses as being very good to good (Table 5). The fact that, in the meantime, 49 participants of the Professional Association of German Surgeons attained the overall certificate documenting attendance of all four courses demonstrates the high acceptance of the curricular concept for teaching minimally invasive surgery skills through the combination of live surgery, lectures, and practical training on a bio simulator.

**Table 5:** **Number of participants and their evaluation of the course content by grades**.

	2012 1st H-Y	2012 2nd H-Y	2013 1st H-Y	2013 2nd H-Y	2014 1st H-Y	2014 2nd H-Y	2015 1st H-Y	2015 2nd H-Y	2016 1st H-Y	*n*
Course 1 *n*	17	21	15	24	24	24	24	23	24	196
Course 1 grade	1.90	1.89	1.33	1.64	1.75	1.43	1.69	1.77	1.81	
Course 2 *n*	18	24	25	24	22	24	23	24	24	184
Course 2 grade	1.29	1.77	1.96	2.02	2.16	1.96	1.71	1.69	2.13	
Course 3 *n*	11	14	19	20	21	19	24	24	21	173
Course 3 grade	1.5	1.46	1.56	1.43	1.61	1.50	2.15	2.14	1.83	
Course 4 *n*	14	18	24	23	20	17	24	24	24	188
Course 4 grade	2.0	2.0	1.83	2.02	1.96	1.75	2.42	2.38	1.94	
										Total *n* 741

*n, number of participants; 1st H-Y, first half-year; 2nd. H-Y, second half-year*.

*Grade = very good (1)*.

*Grade = good (2)*.

*Grade = fair (3)*.

## Discussion

In 2011, the German surgery societies adopted the “laparoscopic surgery curriculum” concept for simulation-based training in laparoscopic surgery. The curricular structure provides for four 2-day training courses with an interrelated, tiered content. The courses are designed to be attended in parallel to the normal specialist surgical training program. The course content is imparted based on live operations, lectures, and exercises carried out on bio simulators. In collaboration with the Professional Association of German Surgeons, 36 courses have, in the meantime, been held at the Vivantes Endoscopic Training Center in Berlin with a total of 741 participants. The courses were evaluated by attendees as being very good and good, i.e., trainees believed they had benefited from the courses. The advantage of this course concept is its direct relevance to the clinical setting with regular facilities for transmission of live operations. This also provides for close supervision by experienced surgeons in minimally invasive surgery. The dedicated training center has a training capacity for 24 trainees. Exercises carried out on biological specimens from the abattoir or supermarket permit intensive training, as resources are not limited. In a systematic review Zendejas et al. (7) demonstrated that laparoscopic techniques can be learned more effectively in a simulation-based training course compared with when learning such techniques only during clinical training.

The skills learned in simulation-based training courses can be immediately applied for the patient in the operating room (12–15). Hence, simulation-based training helps to master the learning curve in minimally invasive surgery and enhance conduct of minimally invasive surgical procedures during the learning curve. Therefore, it is urgently recommended that young surgeons in training participate in such simulation-based courses. Bio simulators, which are used for practicing surgical skills on organ models in the pelvic trainer with standard video-endoscopic equipment, are currently the most cost-effective option. As such, the satisfaction ratings reported by course participants are very high. The positive evaluation by the course attendees, thus, attests to the successful implementation of the scientifically based “laparoscopic surgery curriculum” course concept.

In summary, it can be stated that, participation in the curricular-structured courses in parallel to the normal specialist surgical training program helps to master the learning curve in minimally invasive surgery with simulation-based training and, accordingly, has been evaluated as being very positive by the young surgeons. As consequence, the implementation of such structured educational programs in laparo-endoscopic surgery in every surgical institution performing laparo-endoscopic surgery must be underlined.

## Author Contributions

All authors are actively involved for many years in the “laparoscopic surgery curriculum.”

## Conflict of Interest Statement

The authors declare that the research was conducted in the absence of any commercial or financial relationships that could be construed as a potential conflict of interest.
